# The Incubation of 13α,17-Dihydroxystemodane with *Cephalosporium aphidicola*

**DOI:** 10.3390/molecules17021744

**Published:** 2012-02-09

**Authors:** Braulio M. Fraga, Ricardo Guillermo, Melchor G. Hernández, María C. Chamy, Juan A. Garbarino

**Affiliations:** 1 Instituto de Productos Naturales y Agrobiología, C.S.I.C., Avda. Astrofísico F. Sánchez 3, La Laguna, Tenerife, Canary Islands, 38206, Spain; 2 Instituto Universitario de Bioorgánica “Antonio González”, Departamento de Química Orgánica, Universidad de La Laguna, Tenerife, 38206, Spain; 3 Departamento de Química, Universidad Andrés Bello, Viña del Mar, Chile; 4 Departamento de Química, Universidad Técnica Federico Santa María, Casilla-110V, Valparaiso, Chile

**Keywords:** *Cephalosporium aphidicola*, biotransformations, diterpenes, stemodane

## Abstract

The biotransformation of 13α,17-dihydroxystemodane (**3**) with the fungus *Cephalosporium aphidicola *afforded 13α,17,18-trihydroxystemodane (**4**), 3β,13α,17-tri-hydroxystemodane (**5**), 13α,17-dihydroxy-stemodan-18-oic acid (**6**), 3β,11β,13α,17-tetra-hydroxystemodane (**7**), 11β,13α,17,18-tetrahydroxystemodane (**8**) and 3β,13α,17,18-tetra-hydroxystemodane (**9**). The hydroxylation at C-18 of the substrate points to a biosynthetically-directed transformation, because aphidicolin (**2**) is hydroxylated at this carbon. However, the C-3(β) and C-11(β) hydroxylations seem to indicate a xenobiotic biotransformation.

## 1. Introduction

Microbiological transformations can be divided into two groups: xenobiotic biotransformations, in which the substrate is strange to the transforming organism, and biosynthetically-directed transformations, also known as “analogue biosynthesis”, in which the substrate possesses a structure analogous to a natural biosynthetic intermediate found in the microorganism [1,2]. We have carried out both types of biotransformations using the fungi *Mucor plumbeus* [3] and *Gibberella fujikuroi* [4] respectively. Now, in this work we have used another fungus, *Cephalosporium aphidicola*, which occupies the borderline between the xenobiotic and biosynthetically-directed biotransformations, because it achieves both [5,6,7,8].

**Scheme 1:**
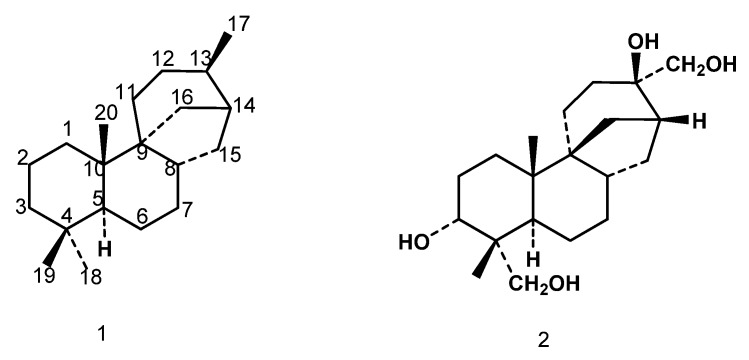
Stemodane (**1**) and aphidicolin (**2**).

Diterpenes with a stemodane skeleton (*i.e*., **1**) have a structural similarity with aphidicolin (**2**), an antiviral substance and a inhibitor of DNA polymerase, which was isolated from *C. aphidicola* [9], although the C/D ring junctions and the configuration at C-13 in stemodanes and aphidicolanes are different (Scheme 1). Thus, biotransformations of stemodane diterpenes, stemodin and stemodinone, with this fungus, have been carried out [10,11]. Some of us had isolated 13α,17-dihydroxystemodane (**3**), and analogous compounds of this type, from *Stemonia chilensis*, a plant that grows in the littoral zone of central Chile [12]. This compound had been incubated with *M. plumbeus* [13], a fungus used in xenobiotic biotransformations.

## 2. Results and Discussion

The microbiological transformation of 13α,17-dihydroxystemodane (**3**) with the fungus *C. aphidicola* afforded 13α,17,18-trihydroxystemodane (**4**), 3β,13α,17-trihydroxystemodane (**5**), 13α,17-dihydroxystemodan-18-oic acid (**6**), 3β,11β,13α,17-tetrahydroxystemodane (**7**), 11β,13α,17,18-tetra-hydroxystemodane (**8**) and 3β,13α,17,18-tetrahydroxystemodane (**9**). Some of these metabolites were obtained as their acetates by acetylation of chromatographic fractions containing them.

The metabolite **4** showed in the HRMS spectrum the ion of higher mass at *m/z* 304.2407, formed from the molecular ion by loss of water, which indicated its molecular formula, C_20_H_34_O_3_. Thus, a new oxygen had been introduced in the molecule during the incubation. In the ^1^H-NMR spectrum the signal of a new AB system appears at δ 3.11 and 3.35 (1H each, d, *J =* 11 Hz). ^13^C-NMR spectrum showed a new signal at δ 72.6 (t). This last value is characteristic of an equatorial -CH_2_OH group at C-4 [14,15], confirmed in the HMBC experiment with correlations of H-18 with C-3, and H-19 with C-3, C-5 and C-18. Therefore, the structure of this compound was determined as 13α,17,18-trihydroxystemodane (**4**).

**Scheme 2:**
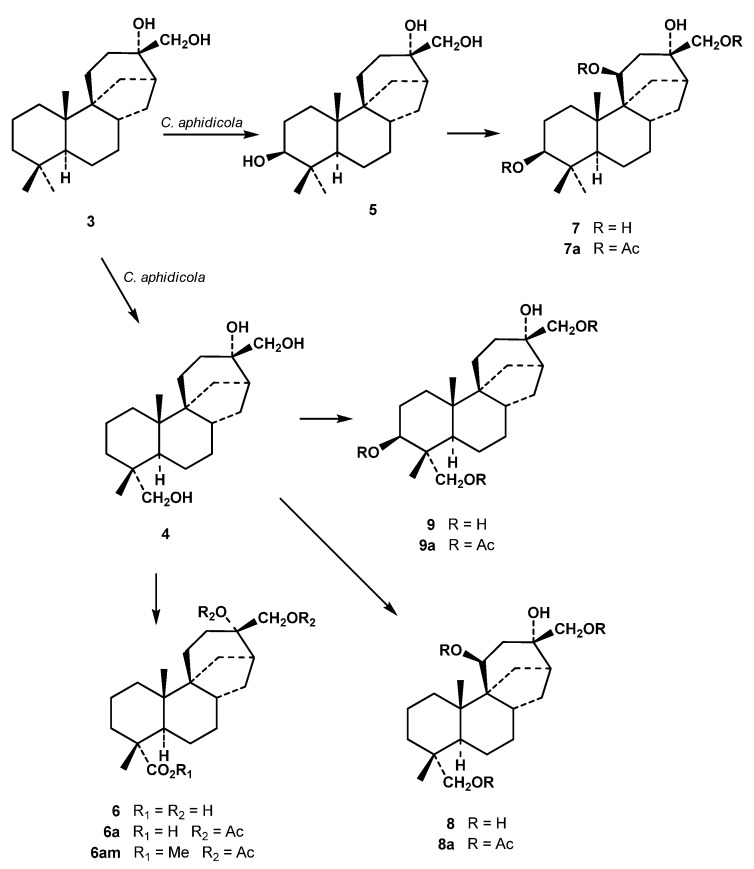
Biotransformation of **1** by *Cephalosporium aphidicola.*

Compound **6** was obtained as its diacetate **6a** by acetylation of the fractions containing it. The molecular formula of **6a** was determined as C_24_H_38_O_6_ considering HRMS data. Therefore, the substrate had gained two oxygens and lost two hydrogens during the fermentation. The two oxygens must form a part of an acid, because in the ^13^C-NMR spectrum a new signal was detected at δ 182.0, typical of this group, while the disappearance of a methyl signal was noted. The presence of this new group was confirmed because **6a** formed a methyl ester (compound **6am**) by treatment with diazomethane. We considered that the C-18 acid must be formed by oxidation of the corresponding alcohol in **4** (Scheme 2), and consequently assigned the structure of 13α,17-dihydroxystemodan-18-oic acid (**6**) to the original metabolite formed in the biotransformation.

**Table 1:** ^13^C-NMR data of compounds **3**, **4**, **6a**, **6am**, **8a** and **9a ** (CDCl_3_).

Position	3	4	6a	6am	8a	9a
1	36.2	35.8	35.7	35.9	36.0 ^a^	33.5
2	18.8	18.1	18.1	18.7	17.6	23.2
3	41.8	35.2	36.8	36.8	36.3 ^b^	74.6
4	33.2	37.6 ^a^	47.2	47.7	36.5	40.8
5	47.2	40.4	41.6	41.9	41.1	39.5
6	22.2	22.0	24.6	24.6	22.1	21.6
7	36.6	36.2	36.7	36.7	34.6	36.1
8	37.3	37.2	37.7	37.7	33.6	37.2
9	50.7	50.8	50.6	50.6	55.0	50.9
10	38.5	38.3 ^a^	37.9	38.0	39.1	38.1
11	27.2	27.3	26.0	26.1	71.7	27.1
12	28.1	28.2	27.0	27.1	36.6^b^	28.1
13	74.3	74.2	84.4	84.5	74.0	74.6
14	40.4	40.5	39.1	39.1	40.3	41.0
15	37.4	37.4	35.2	35.2	35.5 ^a^	37.2
16	29.7	29.8	29.8	29.9	28.4	29.4
17	68.0	68.1	64.7	64.7	69.9	69.8
18	34.5	72.6	182.0	179.6	73.3	65.9
19	22.8	18.6	17.7	17.8	18.5	13.9
20	18.8	19.6	19.0	19.0	20.6	19.4

^a,b^ These values can be interchanged

Compound **8** was obtained as its triacetate **8a**, the mass spectrum of which showed a peak at *m/z* 446.2687 formed from the molecular ion by loss of water. Thus, its molecular formula was determined as C_26_H_40_O_7_. Its NMR spectra showed two –CH_2_OAc groups, one corresponding to the acetylated C-17 alcohol of the substrate, and the other, formed by acetylation of an hydroxyl group introduced in the incubation, resonates at δ_H_ 3.61 and 3.98 (each 1H, d, *J =* 10.8 Hz) and at δ_C_ 73.3 (t). These signals are characteristic of an equatorial acetoxymethylene group at C-4 [14,15]. Other signals observed in the spectra of **8a** were those of an oxymethine group at δ_H_ 5.36 (t, *J =* 7.6 Hz) and δ_C_ 71.7(d). These chemical shifts and couplings were analogous to those of an 11β-acetoxy derivative described in the biotransformation of the substrate **3** by *M. plumbeus* [13]. The HMBC experiment of **8a** confirmed these assignments with the following crosspeaks: H-11 with C-8; H-18 with C-3, C-4 and C-5; H-19 with C-3, C-4, C-5 and C-18; H-20 with C-1, C-5, C-9 and C-10. Thus, the structure 11β,13α,17,18-tetrahydroxystemodane was assigned to the metabolite **8** (Scheme 2) obtained in this fermentation. 

Acetylation and chromatography of the fractions containing **9** led to the triacetate **9a**, which is an isomer of **8a**. In addition to the signals of the 17-CH_2_OAc, in the ^1^H-NMR spectrum of the triacetate **8a** another acetoxymethylene group was detected at δ_H_ 3.67 and 3.88 (each 1H, d, *J =* 11.6 Hz) and δ_C_ 65.9(t), which was assigned to C-4 with an α-equatorial configuration. Thus, in the HMBC experiment the main observed correlations were: H-3 with C-1, C-2, C-4, C-18 and C-19; H-18 with C-3, C-5 and C-19; H-19 with C-3, C-4, C-5 and C-18. These crosspeaks also showed that another acetoxy group was located at C-3, with resonances of this oxymethine at δ_C_ 74.6 and δ_H_ 4.78 (dd, *J =* 11.7 and 4.1 Hz). The coupling constant of this geminal proton to this acetoxy group indicated a β-equatorial configuration for this oxygenated function. Consequently, the structure of the original alcohol formed in the feeding was determined as 3β,13α,17,18-tetrahydroxystemodane (**9**).

Compounds **4** and **6–9** are described here for the first time, whilst 3β,13α,17-trihydroxystemodane (**5**) and 3β,11β,13α,17-tetrahydroxystemodane (7) were already isolated from the biotransformation of **3** with *M. plumbeus *[13].

## 3. Experimental

### 3.1. General Procedures

^1^H- and ^13^C-NMR spectra were recorded at 500.13 and 125.03 MHz, respectively, in a Bruker AMX-500 spectrometer. Mass spectra were taken at 70 eV (probe) in a Micromass Autospec spectrometer. HPLC was performed using a Beckman System Gold 125P. Purification by HPLC was achieved using a silica gel column (Ultrasphere Si 5 μm, 10 × 250 mm). Dry column chromatography was carried out on silica gel Merck 0.040–0.063 mm.

### 3.2. Microorganism

The fungus strain *Cephalosporium aphidicola* IMI 68689 was a gift from Prof. J. R. Hanson, School of Chemistry, University of Sussex, UK.

### 3.3. Incubation of ***3***

*C. aphidicola* was grown in shake culture at 25 °C, in 20 conical flasks (250 mL), each containing 100 mL of a sterile medium comprising (per L) glucose (80 g), NH_4_NO_3_ (0.48 g), KH_2_PO_4_ (5 g), MgSO_4_ (1 g), and trace elements solution (2 mL). The trace elements solution contained (per 100 mL) Co(NO_3_)_2_ (0.01 g), CuSO_4_ (0.015 g), ZnSO_4_ (0.16 g), MnSO_4_ (0.01 g), (NH_4_)_6_Mo_7_O_24_ (0.01 g). 13α,17-Dihydroxystemodane (**3**, 230 mg) dissolved in EtOH (4.5 mL) was evenly distributed in 20 flasks after one day growth. After a further eight days, the fermentation was harvested. The mycelium was filtered and the culture filtrate was extracted with EtOAc. The extract was dried over Na_2_SO_4_ and the solvent evaporated to yield a residue (740 mg) that was chromatographed on a silica gel column in a petroleum ether-EtOAc gradient, to afford starting material **3** (30 mg), 13α,17,18-tri-hydroxystemodane (**4**, 4 mg), 3β,13α,17-trihydroxystemodane (**5**, 6 mg), 13α,17-dihydroxy-stemodane-18-oic acid (**6**) (1 mg), 3β,11β,13α,17-tetrahydroxystemodane (**7**, 2 mg), 11β, 13α,17,18-tetrahydroxystemodane (**8**, 1,5 mg) and 3β,13α,17,18-tetrahydroxystemodane (**9**, 3 mg).

*13α,17,18-Trihydroxystemodane* (**4**). ^1^H-NMR (CDCl_3_): δ 0.83 (3 H, s, H-19), 1.01 (3H, s, H-20), 1,60 (1H, dd, *J =* 11,4 and 2,4 Hz, H-5), 1.79 (2H, br s, H-16), 1.89 (1 H, ddt, *J =* 13.2, 7.4 and 3.1 Hz, H-7β), 2.15 (1H, br s, W_1/2_ = 16 Hz, H-14), 3.11 and 3.35 (each 1H, d, *J =* 11.0 Hz, H-18), 3.38 and 3.45 (each 1H, d, *J =* 10.9 Hz, H-17). EIMS *m/z* (rel. int.): 304 [M−H_2_O]^+^ (2), 291 (66), 286 (17), 273 (100), 255 (54), 230 (8), 215 (12), 206 (18), 203 (40), 173 (29), 159 (22). Found [M−H_2_O]^+^ at *m/z* 304.2407. C_20_H_32_O_2_ requires 304.2402.

*13α,17-Dihydroxystemodane-18-oic acid* (**6**). Obtained as its diacetate **5a** by acetylation and chromatography of the fractions containing it, ^1^H-NMR (CDCl_3_): δ 1.00 (3H, s, H-20), 1.25 (3H, s, H-19), 1.87 (2H, m, H-1 and H-16), 1.97 (1H, dd, *J =* 13.7 and 5.6 Hz, H-11), 2.05 and 2.06 (each 3H, s), 2.16 (1H, dd, *J =* 12.1 and 2.0 Hz, H-5), 2.80 (1 H, br t, *J =* 7.0 Hz, H-14), 4.36 and 4.51 (each 1H, d, *J =* 12.2 Hz, H-17). EIMS *m/z* (rel. int.): 360 [M−C_2_H_4_O_2_]^+^ (5), 318 (33), 305 (16), 300 (24), 285 (12), 277 (19), 255 (12), 239 (7), 220 (22), 204 (17), 184 (35), 159 (20). Found [M-C_2_H_4_O_2_]^+^ at *m/z* 360.2291. C_22_H_32_O_4_ requires 360.2301. *Acetate methyl ester* (**5am**). ^1^H-NMR (CDCl_3_): δ 1.00 (3H, s, H-20), 1.25 (3H, s, H-19), 2.04 and 2.06 (each 3H, s), 2.14 (1H, dd, *J =* 12.1 and 2.2 Hz, H-5), 2.81 (1 H, br t, *J =* 7.0 Hz, H-14), 3.65 (3H, s, -OMe), 4.35 and 4.51 (each 1H, d, *J =* 12.1 Hz, H-17). EIMS *m/z* (rel. int.): 374 [M-C_2_H_4_O_2_]^+^ (9), 332 (82), 314 (52), 299 (24), 277 (38), 255 (75), 239 (27), 234 (44), 220 (17), 199 (15), 185 (27). Found [M−C_2_H_4_O_2_]^+^ at *m/z* 374.2456. C_23_H_34_O_4_ requires 374.2457.

*11α,13α,17,18-Tetrahydroxystemodane* (**8**). Obtained as its triacetate **8a** from the fractions containing it, ^1^H-NMR (CDCl_3_): δ 0.89 (3H, s, H-19), 1.02 (3 H, s, H-20), 1.72 (2H, m, H-1 and H-15), 2.00, 2.06 and 2.08 (each 3H, s), 2.10 (1 H, m, H-14), 2.33 (1H, m, H-8) 3.61 and 3.98 (each 1H, d, *J =* 10.8 Hz, H-18), 3.96 and 3.99 (each 1 H, d, J 11.2 Hz, H-17), 5.36 (1H, t, *J =* 7.6 Hz, H-11). EIMS *m/z* (rel. int.): 446 [M−H_2_O]^+^ (1), 404 (2), 386 (33), 371 (6), 344 (23), 331 (21), 313 (10), 276 (14), 274 (13), 253 (17), 215 (76), 201 (14), 189 (47), 129 (100). Found [M−H_2_O]^+^ at *m/z* 446.2687. C_26_H_38_O_6_ requires 446.2668.

*3β,13α,17,18-Tetrahydroxystemodane* (**9**). Obtained as its triacetate **9a** from the fractions containing it, ^1^H-NMR (CDCl_3_): δ 0.90 (3 H, s, H-19), 1.03 (3H, s, H-20), 1.30 (2H, m, H-6), 1.74 (1H, m, H-8), 1.91 (2H, m, H-7 and H-16), 2.02, 2.07 and 2.09 (each 3H, s), 2.13 (1H, br t, *J =* 7.0 Hz, H-14), 3.67 and 3.88 (each 1H, d, J 11.6 Hz, H-18), 3.91 and 4.00 (each 1H, d, *J =* 11.3 Hz, H-17), 4.78 (1H, dd, J 11.7 and 4.1 Hz, H-3). EIMS *m/z* (rel. int.): 404 [M−C_2_H_4_O_2_]^+^ (6), 391 (24), 331 (9), 326 (29), 311 (14), 284 (16), 271 (14), 269 (12), 266 (49), 251 (39), 223 (17), 197 (14), 186 (100). Found [M−C_2_H_4_O_2_]^+^ at *m/z* 404.2544. C_23_H_35_O_5_ requires 404.2563.

## 4. Conclusions

Several conclusions can be deduced from the microbiological transformation of 13α,17-dihydroxystemodane (**3**) with *C. aphidicola*:

1. The hydroxylations produced in the substrate **3** by this fungus occurred at C-3(β), C-11(β) and C-18.
2. The hydroxylation at C-18 points to a biosynthetically-directed transformation, since aphidicolin (2) is also hydroxylated at this carbon. This position was also functionalized in the biotransformation of stemodine and stemodinone with *C. aphidicola* [10,11]. However, the C-3(β) and C-11(β) hydroxylations, also observed in the incubation of **3**, seem to indicate a xenobiotic biotransformation. These hydroxylations were also observed in the feeding of **3** with *M. plumbeus* [13], a fungus used in the latter type.
3. The oxidation of C-18 to acid level, as occurs in the formation of **6** from **4**, has now been observed for the first time in a biotransformation with *C. aphidicola*.
4. It is probable that the formation of **9** only occurs from **4**, and not from **5**. Thus the hydroxylation of the C-18 methyl in **5** to form **9** could be inhibited by the presence of the equatorial β-hydroxyl group at C-3 (Scheme 2). In aphidicolin biosynthesis has been noted that an axial α-hydroxyl at C-3 blocks the hydroxylation of C-18 [8], whilst in gibberellin biosynthesis has been observed that an equatorial 3α-OH inhibits hydroxylation of C-19 [16].

## References

[1] Thiericke R., Rohr J. (1993). Biological variation of microbial metabolites by precursor-directed biosynthesis. Nat. Prod. Rep..

[2] Hanson J.R. (1992). The microbiological transformation of diterpenoids. Nat. Prod. Rep..

[3] Fraga B.M., González-Vallejo V., Guillermo R. (2011). On the biotransformation of *ent*-trachylobane to *ent*-kaur-11-ene diterpenes. J. Nat. Prod..

[4] Fraga B.M., González-Vallejo V., Guillermo R., Díaz L.N. (2010). Biotransformation of 7α-hydroxy- and 7-oxo-*ent*-atis-16-ene derivatives by the fungus *Gibberella fujikuroi*. Phytochemistry.

[5] Hanson J.R., Nasir H., Parvez A. (1996). The hydroxylation of testosterone and some relatives by *Cephalosporium aphidicola*. Phytochemistry.

[6] Atta-ur-Rahman, Yaqoob A., Farooq A., Anjun S., Fahim A., Choudhary M.I. (1998). Fngal transformation of (*1R*, *2S*, *5R*)-(−)-menthol by Cephalosporium aphidicola. J. Nat. Prod..

[7] Hanson J.R., Jarvis A.J., Ratcliffe A.H. (1992). Biotransformation of some aphidicolane derivatives by *Cephalosporium aphidicola*. Phytochemistry.

[8] Hanson J.R., Jarvis A.J., Laboret F., Takahashi J. (1995). The incubation of 3α,16β-dihydroxyaphidicolane with *Cephalosporium aphidicola*. Phytochemistry.

[9] Dalziel W., Hesp B., Stevenson K.M., Jarvis J.A.J. (1973). The structure and absolute configuration of the antibiotic aphidicolin: A tetracyclic diterpenoid containing a new ring system. J. Chem. Soc. Perkin Trans. I.

[10] Badria F.A., Hufford C.D. (1991). Microbial transformations of stemodin, a *Stemodia* diterpene. Phytochemistry.

[11] Hanson J.R., Reese P.B., Takahashi J.A., Wilson M.R. (1994). Biotransformation of some stemodane diterpenoids by *Cephalosporium aphidicola*. Phytochemistry.

[12] Chamy M.C., Piovano M., Garbarino J.A., Gambaro V. (1991). Stemodane diterpenes from *Stemodia chilensis*. Phytochemistry.

[13] Fraga B.M., Guillermo R., Hernández M.G., Chamy M.C., Garbarino J.A. (2004). Biotransformation of two stemodane diterpenes by *Mucor plumbeus*. Tetrahedron.

[14] González A.G., Arteaga J.M., Bretón J.L., Fraga B.M. (1977). Five new labdane diterpene oxides from *Eupatorium jhanii*. Phytochemistry.

[15] González A.G., Fraga B.M., Hernández M.G., Hanson J.R. (1981). The ^13^C NMR spectra of some *ent*-18-hydroxy-kaur-16-enes. Phytochemistry.

[16] Fraga B.M., González A.G., Hanson J.R., Hernández M.G. (1981). The microbiological transformation of some *ent*-3β-hydroxykaur-16-enes by *Gibberella fujikuroi*. Phytochemistry.

